# 523 Retrospective Application of Guidelines for Referral to Palliative Care for Persons Who Have Been Burned

**DOI:** 10.1093/jbcr/irad045.120

**Published:** 2023-05-15

**Authors:** Daniel Grossoehme, Brianna Bish, Richard Lou, Anjay Khandelwal, Miraides Brown, Carrie Brown, Esther Teo, Sarah Friebert

**Affiliations:** Akron Children's Hospital, Akron, Ohio; Akron Children's Hospital, Kent, Ohio; Akron Children's Hospital, Akron, Ohio; Akron Children's Hospital, Akron, Ohio; Akron Children's Hospital, Akron, Ohio; University of Arkansas for Medical Sciences, Little Rock, Arkansas; University of Arkansas for Medical Sciences, Little Rock, Arkansas; Akron Children's Hospital, Akron, Ohio; Akron Children's Hospital, Akron, Ohio; Akron Children's Hospital, Kent, Ohio; Akron Children's Hospital, Akron, Ohio; Akron Children's Hospital, Akron, Ohio; Akron Children's Hospital, Akron, Ohio; University of Arkansas for Medical Sciences, Little Rock, Arkansas; University of Arkansas for Medical Sciences, Little Rock, Arkansas; Akron Children's Hospital, Akron, Ohio; Akron Children's Hospital, Akron, Ohio; Akron Children's Hospital, Kent, Ohio; Akron Children's Hospital, Akron, Ohio; Akron Children's Hospital, Akron, Ohio; Akron Children's Hospital, Akron, Ohio; University of Arkansas for Medical Sciences, Little Rock, Arkansas; University of Arkansas for Medical Sciences, Little Rock, Arkansas; Akron Children's Hospital, Akron, Ohio; Akron Children's Hospital, Akron, Ohio; Akron Children's Hospital, Kent, Ohio; Akron Children's Hospital, Akron, Ohio; Akron Children's Hospital, Akron, Ohio; Akron Children's Hospital, Akron, Ohio; University of Arkansas for Medical Sciences, Little Rock, Arkansas; University of Arkansas for Medical Sciences, Little Rock, Arkansas; Akron Children's Hospital, Akron, Ohio; Akron Children's Hospital, Akron, Ohio; Akron Children's Hospital, Kent, Ohio; Akron Children's Hospital, Akron, Ohio; Akron Children's Hospital, Akron, Ohio; Akron Children's Hospital, Akron, Ohio; University of Arkansas for Medical Sciences, Little Rock, Arkansas; University of Arkansas for Medical Sciences, Little Rock, Arkansas; Akron Children's Hospital, Akron, Ohio; Akron Children's Hospital, Akron, Ohio; Akron Children's Hospital, Kent, Ohio; Akron Children's Hospital, Akron, Ohio; Akron Children's Hospital, Akron, Ohio; Akron Children's Hospital, Akron, Ohio; University of Arkansas for Medical Sciences, Little Rock, Arkansas; University of Arkansas for Medical Sciences, Little Rock, Arkansas; Akron Children's Hospital, Akron, Ohio; Akron Children's Hospital, Akron, Ohio; Akron Children's Hospital, Kent, Ohio; Akron Children's Hospital, Akron, Ohio; Akron Children's Hospital, Akron, Ohio; Akron Children's Hospital, Akron, Ohio; University of Arkansas for Medical Sciences, Little Rock, Arkansas; University of Arkansas for Medical Sciences, Little Rock, Arkansas; Akron Children's Hospital, Akron, Ohio

## Abstract

**Introduction:**

Palliative Care (PC) is a limited resource, requiring allocation to those most likely to benefit. One currently underserved population is persons sustaining burns, as these injuries may severely limit quality of life, psychosocial and physical health. Additionally, treatment decisions must frequently be made on an emergent basis on behalf of someone unable to participate in decision making. PC services could benefit patients and caregivers substantially, yet only 2% of persons who have been burned are referred to PC. We developed empirically-derived referral criteria; the purpose of this study was to quantify the sensitivity, specificity, and predictive value of the criteria when retrospectively applied.

**Methods:**

Retrospective review of regional burn center admissions (1/1/2019-1/31/2022) to determine adherence to criteria indicating referral to PC should be made or considered. Descriptive statistics characterizing the sample and criteria performance were calculated.

**Results:**

N=388 admissions occurred study period (29.9% female; mean(SD) age=39.9 (24.1) years. N=28 persons were referred to PC; n=27 met consult criteria, and n=1 met consider criteria. Criteria for PC consultation indicated referral for n=62, and had 0.96 sensitivity, 0.90 specificity, 0.44 positive predictive value, 1.00 negative predictive value, 0.91 total accuracy. Criteria for considering PC consultation were indicated for n=181 and had 1.00 sensitivity, 0.45 specificity, 0.01 positive predictive value, 1.00 negative predictive value, 0.45 total accuracy.

**Conclusions:**

Criteria for PC consultation were sensitive and specific, though the positive predictive value was low. Criteria for consideration show low values; however, documentation did not reveal whether referral was considered or not. Presence of comorbidities was the most commonly-met criterion for referral consideration. Despite the robust sample size, data from a single site may have limited generalizability. Criteria have promising characteristics, may be used as basis for quality improvement efforts, and merit ongoing study and revision to improve predictive ability.

**Applicability of Research to Practice:**

These criteria advance clinical practice by providing guidance on identifying persons most likely to benefit from specialist PC.

